# Gender roles, generational changes and environmental challenges: an intersectional interpretation of perceptions on healthy diets among iTaukei women and men in Fiji

**DOI:** 10.1017/S1368980022001677

**Published:** 2022-11

**Authors:** Briar Louise McKenzie, Gade Waqa, Ashleigh Chanel Hart, Anasaini Moala Silatolu, Anna Palagyi, Robyn Norton, Rachael McLean, Jacqui Webster

**Affiliations:** 1 The George Institute for Global Health, University of New South Wales, Sydney, NSW 2042, Australia; 2 C-POND, Fiji Institute for Pacific Health Research, Fiji National University, Suva, Fiji; 3 The George Institute for Global Health, Imperial College London, London, UK; 4 Department of Preventive and Social Medicine, University of Otago, Dunedin, New Zealand

**Keywords:** Diet, Disease, Fiji, Nutrition interventions, Food policy, Gender, Intersectionality

## Abstract

**Objective:**

To investigate perceptions of iTaukei Fijian women and men around diet and the ability to consume a healthy diet.

**Design:**

Six focus groups were conducted with women and men separately. Six to ten women and men participated in each group. Discussions were recorded, transcribed, translated and thematically analysed. Themes were mapped to an intersectionality framework to aid interpretation.

**Setting:**

Four villages in Viti Levu, Fiji.

**Participants:**

Twenty-two women and twenty-four men.

**Results:**

Seven overarching themes were identified, including generational changes in food behaviour, strong-gendered beliefs around food and food provision, cultural and religious obligations around food, the impact of environmental change on the ability to consume a healthy diet, perceptions of the importance of food, food preferences and knowledge. Participants across focus groups identified that it was the ‘duty’ of women to prepare food for their families. However, some women reflected on this responsibility being unbalanced with many women now in the formal workforce. Changes between generations in food preferences and practices were highlighted, with a perception that previous generations were healthier. Power dynamics and external factors, such as environmental changes, were identified by women and men as crucial influences on their ability to eat a healthy diet.

**Conclusion:**

Embedded traditional perceptions of gendered roles related to nutrition were misaligned with other societal and environmental changes. Given factors other than gender, such as broader power dynamics and environmental factors were identified as influencing diet, viewing nutrition-related issues through an intersectional lens is important to inform equitable food policy in Fiji.

Globally, non-communicable diseases are the leading causes of death and are predicted to retain this position for the foreseeable future^(1,2)^. There is a dietary risk component to many non-communicable diseases, with diets high in salt, saturated and trans-fats, sugar and ultra-processed foods associated with increased risks of hypertension, CVD, obesity, diabetes and some cancers^(3)^. Pacific Island countries experience some of the highest rates of non-communicable diseases^(1)^. It is hypothesised that this non-communicable disease burden is in part due to a transition from the more traditional plant- and seafood-based diets to westernised diets characterised by high fat, salt and sugar intakes^(4,5)^.

Fiji is an upper middle-income country and has a population of approximately 900 000 people, spanning around 100 islands^(6)^. Approximately half of the population lives in urban areas^(7)^. Fiji has two main ethnic groups, Indigenous iTaukei Fijians and Indo-Fijians or Fijians with Indian heritage. The burden of disease differs by ethnic group and sex^(8,9)^. iTaukei Fijian men have a higher risk of dying from CVD than iTaukei women, Indo-Fijian women and Indo-Fijian men^(8)^. However, in the overall Fijian population, almost twice the number of women compared to men live with obesity^(10)^. The prevalence of obesity is highest for iTaukei women, compared to iTaukei men and Indo-Fijian women and men^(10,11)^. Additionally, the proportion of type two diabetes attributable to high BMI is greater in the iTaukei Fijian population than in the Indo-Fijian population^(11)^.

Gendered roles and responsibilities can influence food provision and the health of individuals and families^(12–14)^. In many countries, including Fiji, women tend to be responsible for the bulk of the childrearing and household work^(15)^. Additionally, in traditional iTaukei culture, ideas around femininity and masculinity, largely based on Christian ideals, may influence roles around food, with women as carers and nurturers being responsible for food preparing and cooking, while men have the role of head of the family and, therefore, having the first serve of meals^(16)^. It is important to understand the different gendered roles and responsibilities around food, along with understanding what these roles are influenced by (including culture, social systems and religion), in order to establish effective diet interventions and food policy in Fiji to improve health equitably^(17,18)^. The term ‘intersectionality’ acknowledges that aspects of someone’s identity (e.g., gender, ethnicity, religion), interact with each other and interact with how a person experiences social systems and their environment more broadly. An intersectionality framework can then be used to understand the interaction of demographic characteristics, social identities and environmental factors, which can be helpful to inform the development and implementation of more impactful policy^(17,18)^. Given a new Food and Nutrition Security Policy is due to be endorsed in Fiji in 2022, we propose that this research will help inform the type of interventions and support needed to effectively implement the policy equitably.

There is a need to hear the voices of community members to understand their interpretation of a healthy diet and how gender roles and responsibilities and other equity factors are implicit in this. Therefore, the aim of this study was to investigate perceptions around diet and the ability to consume a healthy diet with an intersectional interpretation. Given the differing prevalence of diet-related disease risk factors between the main ethnic groups in Fiji, the focus of this study was within the iTaukei population.

## Methods

### Terminology

We hypothesised that societal and environmental factors would have a greater influence on diet knowledge, attitudes and behaviours, rather than biological (sex) factors. Therefore, the term ‘gender’ has been used throughout this study. However, we acknowledge that gender is not a binary construct, and that we have focussed on only two gender identities within this study (women and men). We did not recruit people with other gender identities, and as such we cannot conflate our findings to a broader spectrum of gender identities.

### Participants and procedure

Six focus groups were conducted with women and men, separately, across four villages in the Central Division of Viti Levu, Fiji. A convenience sample of villages in two rural (*n* 3 focus group discussions (FGD)) and two peri-urban (*n* 3 FGD) were selected based on the local social and geographic knowledge and experience of Fijian members of the research team. Village leaders were approached a week before the planned focus group discussion to seek permission to visit the village and conduct research. On an agreed day and time in early December 2019, two researchers went to the village, where a I sevusevu (a gift) was presented to the village chief or official representative, seeking their consent to conduct research in their village. Potential participants were identified by village health care workers, who approached six to ten women and men to participate who were at home on the day of the focus group discussion. This number of participants per focus group was sought to ensure both diversity of lived experience and productive group discussion^(19)^. In one peri-urban village, men were not available for a focus group during the study period as they were either away from the village working or otherwise unable to participate, and therefore a fourth village, in a rural setting, was approached to participate. Potential participants were eligible to participate if they were aged 18 years or older and lived in the village visited. Participants self-selected which group they participated in (women’s focus group or men’s focus group).

Discussions were held in either English, Fijian or a mixture of both, depending on the preference of the group. Focus groups were conducted in village halls and were attended by two researchers: one researcher facilitated and moderated the group discussions while the other made notes and monitored the process. The researcher who facilitated the discussions is an experienced qualitative researcher based in Fiji, having conducted her PhD via a range of qualitative methods. Before beginning the discussion, information was collected on each participant’s age, gender and the number of people that lived in their household. At the end of the discussion, participants were provided with a voucher for staple foods such as fruits, vegetables and grains, from a nearby supermarket, to the value of twenty Fijian dollars. Focus group duration ranged from 45 to 75 min and discussions were audio-recorded.

### Discussion guide

Discussions were guided by a pre-defined, semi-structured discussion guide (online Supplementary Table 1). Questions were based on knowledge, attitudes and behaviours towards food and healthy diets; additional questions on perceptions of the food environment were asked. The discussion guide was written in English, translated into Fijian and back translated into English to check accuracy. The discussion guide was piloted in English and Fijian, with minor amendments made prior to finalisation.

### Data analysis

**Fig. 1:**
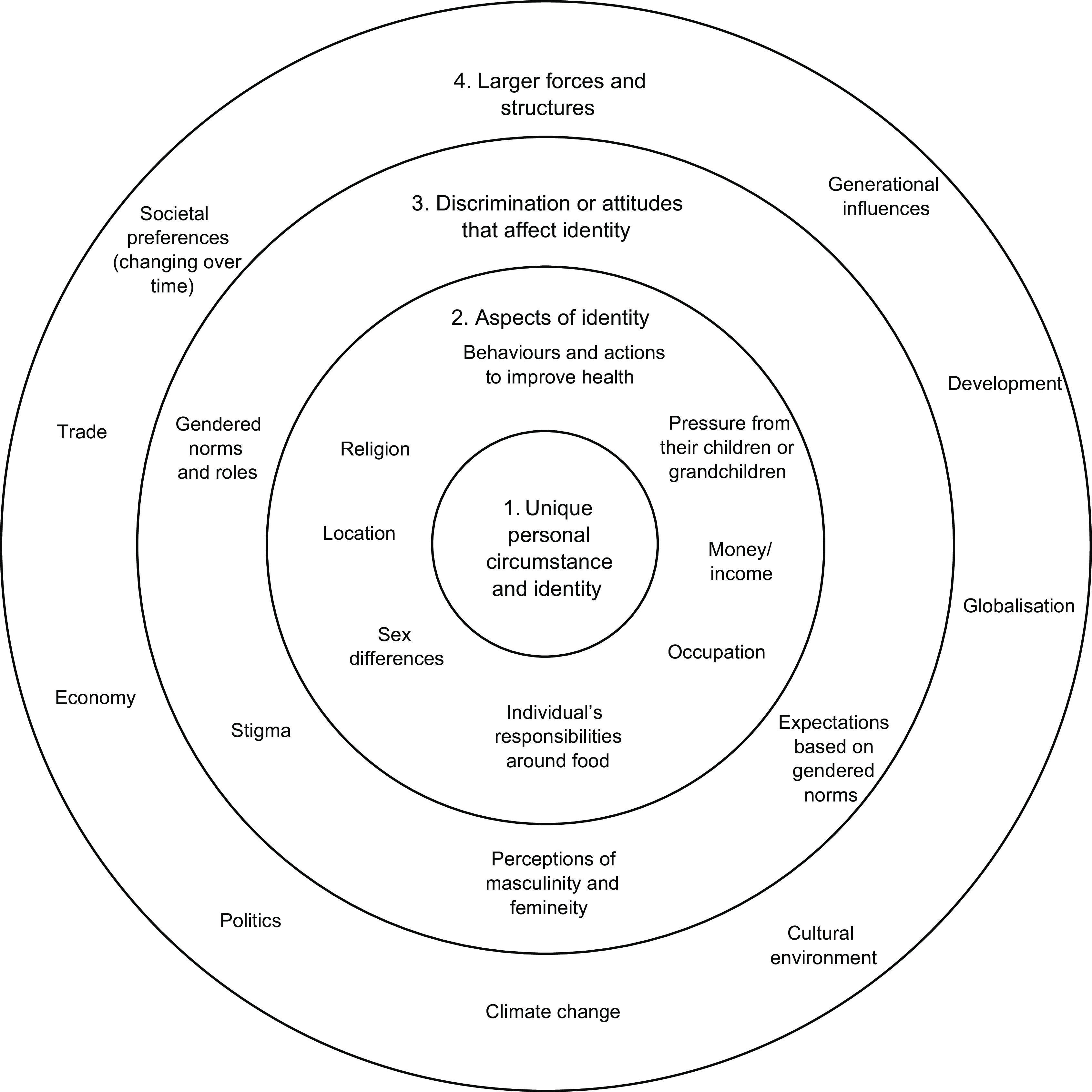
Factors identified within a thematic analysis, mapped to the four levels of the intersectionality wheel^(18,20)^

The recorded discussions were transcribed manually in Fijian and then translated into English. Each transcript was cross-checked for accuracy. NVivo12 was used for data storage and coding. An inductive, thematic approach was used to code the data. Transcripts were independently coded by two researchers, who then compared and discussed identified themes in consultation with other research team members. A coding framework was used to consolidate themes, which was adapted as transcripts were analysed. The identified themes were mapped, deductively to an intersectionality framework, following the World Health Organisation toolkit on the incorporation of intersectional gender analyses into research^(18)^, and visually depicted based on the Canadian Research Institute for the Advancement of Women, Intersectionality Wheel^(20)^ (Fig. 1 shows an interpretation of the Intersectionality Wheel in reference to our study findings). These intersectional factors, viewed in the intersectionality wheel, are either proximal or distal to the individual (shown by the different circles of the wheel), representing that there are many different ‘levels’ or ‘power dynamics’ that influence participants perceived ability to eat a healthy diet. Specifically, the innermost circle represents the individuals unique circumstances/identity, the second circle represents aspects of identity (e.g., sex, gender, age), the third circle represents different types of discrimination or attitudes that impact identity (e.g., perceptions of masculinity or femineity based on heteronormative values), and finally, the outermost circle represents larger forces or structures (e.g., development, politics, economy)^(20)^.

## Results

Twenty-two women (fourteen peri-urban, eight rural) and twenty-four men (ten peri-urban, fourteen rural) participated in the focus group discussions. The mean age of women who participated was 49 years (forty-eight for the peri-urban and fifty-one for the rural focus groups), and for men, it was 44 years (twenty-five for the peri-urban and fifty-eight for the rural focus groups). Women and men had an average of six people in their households (six for peri-urban and five for rural households), Table 1.

**Table 1:** Characteristics of focus group participants

	Peri-urban						Rural							
	Women		Men		All		Women		Men		All		Overall	
Number of participants	14		10		24		8		14		22		46	
	Mean	Range	Mean	Range	Mean	Range	Mean	Range	Mean	Range	Mean	Range	Mean	Range
Age (years)	48	24–67	25	18–42	39	18–67	51	28–69	58	40–76	55	28–76	47	18–76
Number of people in household	6	2–13	6	3–9	6	2–13	6	3–9	5	3–8	5	3–9	6	2–13

Seven overarching themes were identified from across the six focus group discussions (Table 2). Each theme spanned the four levels of the intersectionality wheel (Fig. 1, Table 2), representing the many levels and corresponding power dynamics that influenced the themes and the participants’ perceived ability to eat a healthy diet.

**Table 2:** Themes identified from the focus group discussions, and mapping to intersectional factors

Overarching themes	Subthemes identified in the analysis	Mapping to intersectional factors^(18,20)^	Intersectionality wheel, level^(18,20)^
1. Behaviours around food and reflections on generational changes in food behaviours	Food behaviours	Individual behaviour	1 – Personal circumstance/identity
		Societal pressure (influencing food behaviour)	4 – Larger forces/structures 2 – Aspects of identity
	Actions to improve diets	Individual actions, actions to improve diets of those around them	1- Personal circumstance/identity
	Generational changes in behaviours	Societal pressure/preferences (changing over time)	4 – Larger forces/structures
		Generational influences (in both directions)	4 – Larger forces/structures
		Pressure from children/grandchildren	2 – Aspects of identity
		Development/globalisation	4 – Larger forces/structures
2. Beliefs around food and gendered beliefs	Food beliefs	Perceptions of masculinity and femineity	3 – Discrimination or attitudes that affect identity
		Gendered norms and roles	3 – Discrimination or attitudes that affect identity
		Responsibilities around food	2 – Aspects of identity
		Religion	2 – Aspects of identity
	Gendered beliefs	Stigma	3 – Discrimination or attitudes that affect identity
		Perceptions of masculinity and femineity	3 – Discrimination or attitudes that affect identity
		Gendered norms and roles	3 – Discrimination or attitudes that affect identity
		Responsibilities around food	2 – Aspects of identity
		Religion	2 – Aspects of identity
3. Cultural and religious obligations and influences around food	Food preparation	Perceptions of masculinity and femineity	3 – Discrimination or attitudes that affect identity
		Gender norms and roles	3 – Discrimination or attitudes that affect identity
		Responsibilities around food	2 – Aspects of identity
	Food purchasing	Gendered norms and roles	3 – Discrimination or attitudes that affect identity
		Responsibilities around food	2 – Aspects of identity
		Money	2 – Aspects of identity
	Religious practices involving food	Religion	2 – Aspects of identity
		Expectations based on gendered norms	3 – Discrimination or attitudes that affect identity
		Perceptions of masculinity and femineity	3 – Discrimination or attitudes that affect identity
4. Environmental factors impacting on the ability to achieve a healthy diet	Availability	Development/globalisation	4 – Larger forces/structures
		Geographical location, i.e. immediate surrounds	2 – Aspects of identity
		Climate change	4 – Larger forces/structures
		Money	2 – Aspects of identity
	Access	Development/globalisation	4 – Larger forces/structures
		Geographical location, i.e. immediate surrounds	2 – Aspects of identity
		Money	2 – Aspects of identity
	Environmental context	Geographical location	2 – Aspects of identity
		Climate change	4 – Larger forces/structures
	Importance of being able to grow own food	Geographical location, i.e. immediate surrounds	2 – Aspects of identity
		Climate change	4 – Larger forces/structures
	Impacts of development	Development/globalisation	4 – Larger forces/structures
		Money	2 – Aspects of identity
		Politics	4 – Larger forces/structures
		Economy	4 – Larger forces/structures
		Trade	4 – Larger forces/structures
5. Perceptions around the importance of food and health	Importance of food	Cultural environment	4 – Larger forces/structures
	Perceptions of health	Societal values	4 – Larger forces/structures
		Perceptions of masculinity and femineity	3 – Discrimination or attitudes that affect identity
	Perceptions of healthy food	Cultural environment	4 – Larger forces/structures
		Education	2 – Aspects of identity
6. Food preferences and attitudes and generational changes in preferences	Food preferences and attitudes	Gender norms and roles	3 – Discrimination or attitudes that affect identity
		Societal pressure/preferences (changing over time)	4 – Larger forces/structures
	Generational changes in preferences	Generational influences (in both directions)	4 – Larger forces/structures
		Development/globalisation	4 – Larger forces/structures
		Societal pressure/preferences (changing over time)	4 – Larger forces/structures
7. Knowledge around food, dietary requirements and the relationship between food and disease	Knowledge of food and disease	Education	2 – Aspects of identity
		Environment	2 – Aspects of identity
		Generational influences (in both directions)	4 – Larger forces/structures
	Sources of health information	Education	2 – Aspects of identity
		Environment	2 – Aspects of identity
	Dietary requirements	Sex differences	2 – Aspects of identity
		Occupation	2 – Aspects of identity
		Perceptions of masculinity and femineity	3 – Discrimination or attitudes that affect identity

### Behaviours around food and reflections on generational changes in food behaviours

Discussion around food behaviours focussed on individual and family/friends’ food behaviours, individual actions to improve diets and generational changes in behaviours. Generational changes in behaviour were reflected on by both rural and peri-urban participants, and by women and men, as being key to negative changes in the health of participants and their families. There was discussion around changes in lifestyle, with more women working in the formal (paid) workforce, and that this was changing what their families were eating (i.e. an increased reliance on take-away foods).

*Men are not doing their work… before they used to plant cassava and we always have plenty of it, only the elders used to do that, and we just eat them (the cassava) … Now, the men are sleeping. – W, FG 1, Rural*

*Nowadays, we are only accessed to fast food or take away foods, because we have no time to cook at home, so we go for Pizza and other fast foods. – W, FG 4, Peri-urban*

Women in the rural and peri-urban focus groups also reflected that men were drinking more kava (a herbal depressant) and alcohol, which was impacting how they worked and contributed to the family.

*Just do your plantations and plant vegetables, root crops and there is the sea, streams, and rivers where we can get our food. (Instead) there is too much grog and sleep. –W, FG1, Rural*

*Sometimes we will fight because I always stop him from drinking grog. I have a lot of uncles here, most of the time, they are coming to have grog with him. –W, FG1, Rural*

In the rural focus group, women reflected that men were less often planting crops (for food) or fishing. Instead, they were choosing to purchase food from the local shop when needed. The rural men discussed two key barriers to growth and catching their own foods; (1) that it was now harder to grow the foods that their forefathers used to grow due to changes in the soil and raising sea levels, and (2) that they perceived themselves and others in their village to be less hardworking than previous generations.

*I think one of the causes of the problem as well is that, during this time, the people are not like before - they were hardworking. These days, they wake up at eight or nine o’clock, whereas the elders will work first before breakfast. But people are used to having easy life; wake up in the morning at eight or nine, run to the shop, get one packet of biscuit, and have tea. – M, FG 2, Rural*

This theme covered factors of individual identity (intersectionality (IS) level 1) including individual actions and behaviours around food, aspects of identity (IS level 2) including the living arrangement and influence of family members, and external forces (IS level 4) including social factors stemming into societal pressure and generational change, and the impact of development/globalisation and climate change (Fig. 1, Table 2).

### Beliefs around food and gendered beliefs

Strong gendered beliefs around food and alcohol-related practices were identified. Women highlighted the transition of men towards unhealthy food and dietary practices. Both women and men said that it was the role or ‘*duty*’ of women to prepare food, as well as to look after the health of the family in general.

*Our ancestors didn’t consume kava and grog [alcohol] excessively and now, most of our women are widows because their husbands do not listen to the advice of a woman (their better half). They don’t realise that we (women) are created to assist with providing food from the farm/garden.- W, FG 3, Peri-urban*

The gendered beliefs around meal decisions and food preparation were also shown in terms of relationships. Men, particularly in the rural focus group, reflected that when women prepare food for the family it is a way of them showing their love and care for their husband and family.

*When the food is being prepared nicely by the wife, it will be felt by the man.- M, FG 2,Rural*

Participants also reflected on the fact that men eat first and that women often eat what is leftover, once the rest of the family has served themselves. While there was a consensus across the focus groups that women *should* prepare food, the women’s focus groups agreed that men should no longer get ‘*all the best food*’ – they reflected that women work just as hard as the men, so there should not be a difference for women and men.

*But our culture is that, Men should have more food and us Women, we will eat what is left… But now, for me at home, I will be very honest, my husband and I will eat the same amount of food. Sometimes, I will eat more food than him and he will say ‘hey, you have more’ and I will tell him, ‘I am doing more work than you’. W, FG 1, Rural*

This theme covered aspects of an individual’s identity (IS level 2) including their gender, religion and perceived household roles. It also included aspects of discrimination and attitudes that impact on identity (IS level 3), such gendered norms and roles around food based on perceptions of masculinity and femineity (Fig. 1, Table 2).

### Cultural and religious obligations and influences around food

Strong cultural and religious factors that influenced both food choice and gendered perceptions around food and health were identified. Every focus group discussed their eating practices on a Sunday, a day on which it is common for extended families to come together and share food after church. This is an important part of iTaukei culture, however several of the focus group participants discussed that the food provided at these occasions is unhealthy, and that a lot of food is provided with corresponding large portion sizes. Participants said that leftover food is then re-fried and eaten in the days following.

*‘… one issue that I see which can cause sickness is the amount of fried foods that we are eating…, over here, we do not have a lot of rootcrops. For us, on Sundays, we always have a lot of rootcrops and I always keep the left-over foods. I can fry it today, sometimes I will put it in the container and place it in the Deep and will heat it again. Sometimes, I fry it so that it can be kept for longer period. I know that this will cause sickness but since it is not happening, I keep on doing it.’ W, FG 1, Rural*

*I know that I’m diabetic and I usually separate my food, but on Sundays I can’t… I’m always afraid of eating at home. When they prepare chicken curry… I’m always afraid to eat it so I choose what to cook to eat… I’m afraid to die – W, FG 3, Peri-urban*

This theme spanned aspects of an individual’s identity (IS level 2) including their gender, religion, income and role within the household, and it also included aspects of discrimination and attitudes that impact on identity (IS level 3) (Fig. 1, Table 2).

### Environmental factors impacting on the ability to achieve a healthy diet

Key themes around individuals’ environments, including environmental context (climate change), food availability, food access, impacts of development and the importance of being able to grow their own food, were identified. Climate change, including increased frequency of cyclones and rising sea levels, was reflected on by the rural focus group participants as impacting negatively on their diet.

*During the cyclone, this village was flooded. Seedlings were brought in but we were told that some were planted and some were not. We did not see anything (grow), and people are still buying from the market. - W, FG 1, Rural*

*Before this place was all covered with dalo. Now, sea water is coming this side and that side too. If you are here when the tide is up, this place will all be flooded. – M, FG 2, Rural*

Rural participants identified an increase in the availability of, and access to, processed packaged foods and a decrease in access to traditional and/or home-grown foods. Reasons identified included infrastructure developments (e.g. roads, electricity, shops), climate change with more frequent storms, cyclones and rising sea levels that are impacting people’s ability to grow the foods that their forefathers used to grow, along with more women and men working in the city (Suva).

*…. like the electricity that we have on our roads. Before, our forefathers cross the river using their own lights, but now the electricity is on the road, as well as inside the buses. Before, our forefathers, when they are hungry, they eat from their plantations, what they eat is always healthy. But for us, when there is left over cassava, we put it in the fridge and then we fry it so that we can make use of it. That is not so good. - W, FG 1, Rural*

All participants said that people need to focus more on growing their own foods and fishing to provide food for their families. However, men in one of the peri-urban villages discussed how they were concerned about the pollution and chemicals around the foods that they were growing, and that home-grown foods may not be healthy because of that pollution.

*We do not get a lot of food from the land because there is a lot of chemicals there to make it grow quickly which is not suitable for our body so that we can live longer. That means everything that they do is just to get our lives shorter because there is a lot of chemicals in our food. We should do something like not to put manure on the food and make it grow by itself so that it meets the requirement that our body needs. - M, FG 5, Peri-urban*

Finally, women and men in the rural village focus groups, and women in one of the peri-urban focus group discussions, said that they were worried about sugary snacks available in or around their children’s schools and kindergartens (Kindy). In the rural focus groups, participants said that some of the teachers were selling sugary snacks in the school, and that their children or grandchildren would ask their parents for money to take them to school for this.

*I have a grandchild and he is having toothache every night but, in the morning, we still give him the money because we want the child to go to school. We want to say something to the teachers not to sell those sweets, but we cannot. So that’s it. - W, FG 1, Rural*

*It’s the teachers that are selling there, especially to the Kindy; beans, sweets, lollies, chewing gums, those things. – W, FG 1, Rural*

The women in the peri-urban focus group, said that the food in the schools was healthy, but that people were selling sweets around the schools.

*I have a grandchild that attends [school] and by the time they finish school, those (food hawkers) that sell by the school roadside sell a lot of lollies – W, FG 3, Peri-urban*

These three groups each discussed how this was impacting negatively on their children/grandchildren and that a lot of their children/grandchildren now have dental issues.

Environmental factors impacting on the ability to eat a healthy diet spanned aspects of an individual’s identity (IS level 2) namely their income influencing how/where they accessed food and where they live influencing what food is available. However, the focus was mainly on external forces (IS level 4) that in general were perceived to have negative impacts on the access and availability of healthy foods, including development and globalisation, climate change, politics, economy and trade influences (Fig. 1, Table 2).

### Perceptions around the importance of food and health

Food was perceived as important for both cultural reasons (bringing people together, particularly for religious events) and for providing the body with strength for work. Perceptions on health and how a healthy person would look varied across groups. However, what was consistent was that the signs of good health were much broader than just physical markers like body weight. Both women and men reflected that a ‘healthy woman’ was someone who had a healthy and happy family, who presented herself well and was happy.

*When we see a healthy woman, we will look at her family. The man is looking after her, her children, what she eats, everything she needs. We will see her and say ‘okay, because of her family. – W, FG 2, Rural*

*A healthy woman is one that has a good heart… She protects herself, she grooms herself, and she would greet you when she meets you – W, FG3, Peri-urban*

Women reflected that a healthy man was a man who didn’t drink too much alcohol, who was able to look after himself and who actively provided for his family. Men reflected that you could tell if a man was healthy through how he presented himself and how he took care of his family.

*It will be seen where he lives, his looks, his way of preparing things. How he mixes with people, how he takes care of things in his home, his environment, his family, his children, and grandchildren. – M, FG2, Rural*

Healthy foods were identified as fresh foods that were cooked using traditional methods, for example, most participants said that if they were having a healthy meal, it would be fish cooked in lolo (coconut milk), with vegetables like dalo (Fijian taro). Most stated that unhealthy foods included foods that were fried, were packaged or came from the shops. Although, most of the focus groups discussed that they worried about their children and/or grandchildren as they felt they preferred unhealthy foods.

*For me at home, when we eat a lot of oily food, I always tell them ‘cut down on oily foods’. When there is a lot of lolo [coconut milk] foods, I tell them ‘boil it’. We show the people at home how to prevent sickness that is caused by abusing these foods. - M, FG2, Rural*

Perceptions around the importance of food and health were influenced by education (IS level 2), perceptions of masculinity and femineity (IS level 3) and the cultural environment/societal values (IS level 4) (Fig. 1, Table 2).

### Generational changes in food preferences and attitudes

Across the focus groups, participants said that they preferred locally sourced traditional foods. However, individual preferences were not a strong focus of discussions. Instead, participants focussed on the changing food preferences across generations, including that their children and grandchildren would not eat traditional healthy foods when they are prepared.

*We take it upon ourselves for the children to have foods from the three food groups when they have their lunch in school and the teachers will make sure that the children bring fruits every day, as they are not allowed to eat sweets in school. The children have changed so quickly to have lollies and bongos. When moca (bhajia) is cooked with egg or sausage, the egg or sausage will be eaten and the moca (bhajia) will be left there. They have changed and not like last year. I see that there is a change in terms of the eating pattern, most of the time, children see what the other families are eating and it is not eaten in our house, so they want to eat that food too. That is a big change that I see in our family regarding the quality of food. – W, FG 1, Rural*

This theme of generational changes in food preferences and attitudes included factors of individual identity (IS level 1), gendered norms and roles (IS level 3), values of the society as a collective, development and globalisation and generational influences/changes more broadly (IS level 4) (Fig. 1, Table 2).

### Knowledge around food, dietary requirements and the relationship between food and disease

There was consistently a high level of knowledge around food and what would consist of a healthy diet and healthy food behaviour. Participants reflected on the risk of having a poor diet with diseases like diabetes and heart disease and poor dentition. Participants also knew about the increased nutritional needs for women of reproductive age and the risk of iron deficiency.

*Healthy food like for breakfast, if we have tea with bread or bun, there should be some fruit there. It should have food from the three food groups to keep us healthy, food that gives us iron for our blood and healthy food to make us strong. We should always consider the food that we eat to be healthy so that we can be healthy all the time and will not cause any sickness to us. – W, FG 1, Rural*

*All sickness or health issues are the same eh! If I have kidney problems, ulcer or diabetes, (it’s the) diet (that) determines your healthiness. – W, FG 3, Peri-urban*

All participants discussed that they had support and information from the Ministry of Health and community nurses and information on a healthy diet from television and radio. Both women and men across the settings discussed how they knew what they needed to do to eat healthily; however, it was not always possible to do the right thing.

*I think that we have to change the way we eat. There is a lot of processed food. We should eat the food that lives free, like taro leaves. It is around us, the food that we supposed to eat and then we are going to the shop to buy tinned fish and things like that. I think we should change it. – M, FG 5, Peri-urban*

*For me, when I look at the health of my family, sometimes we eat healthy food which contains everything, we will eat cassava, vegetables and meat. Sometimes, there is nothing at all. – W, FG 1, Rural*

*Sometimes we eat healthy food and sometimes we eat whatever we can afford – M, FG 6, Rural*

Knowledge around food, dietary requirements and the relationship between food and disease was influenced by aspects of an individual’s identity, such as education, physical environment, occupation and sex (IS level 2). It was also influenced by perceptions of masculinity and femineity (IS level 3) and generational changes/influence (IS level 4) (Fig. 1, Table 2).

## Discussion

This is the first study to explore the relationship between gender and diet in a Fijian population using a qualitative approach with an intersectional interpretation. Our findings highlight important gender-related inequities and gendered roles and responsibilities around food among iTaukei Fijians. However, broader power dynamics and external factors, such as environmental change, were identified by both women and men as factors impacting their ability to achieve a healthy diet. The identified influences from external factors (viewed by different levels of the intersectionality wheel) will be helpful when considering the implementation of food-related policies in Fiji.

There were strongly identified gendered roles and responsibilities around food across focus group discussions. Women and men identified that it is the ‘role’ or ‘duty’ of women to cook for their families. Gendered roles and responsibilities were related to religious beliefs and practices, for example, the role of women in preparing food for their extended families on Sundays (to be eaten after attending Church), and practices that dictate men eat first and women eat last as a form of showing respect. Previous studies have discussed at length the role of women in food provision, and how women tend to be the gatekeepers of nutrition for their families^(12–14,21)^. Globally, research shows that women spend substantially more time preparing food, cooking, cleaning and childcaring/rearing than men^(22,23)^. In Fiji, and in many other countries around the world, the proportion of women working in the formal (paid) workforce is increasing^(24)^, and literature has shown a shift to convenience foods that are often highly processed, energy dense but nutrient poor^(21,25)^. Inequities in terms of gendered roles and responsibilities for food provision are therefore misaligned with other societal changes and can have an impact on the health of populations^(25)^. However, this does not mean that women should be ‘kept’ as gatekeepers of nutrition. Instead, it highlights the need for nutrition-related responsibilities to be shared within households and for environments to be conducive to healthy diets, meaning the most convenient options are the healthiest options. For this to occur, there likely needs to be grass-root approaches, including advocacy within and by community groups and feminist organisations, along with the formation of gender-responsive food policy.

A high level of nutrition-related knowledge was evident across focus groups, and this level of knowledge did not appear to differ by gender. However, while participants knew how to eat healthily and knew about the relationship between diet and disease, they reflected that it is not always possible to eat healthily, instead, they eat what they can afford. Fiji’s dietary guidelines focus on three key groups^(26)^: body-building foods (e.g., protein-rich foods like meat and dairy products), energy foods (e.g., carbohydrate-rich foods like rice and bread) and health foods (fruits and vegetables). We found that there was a focus on making sure that families had body building and energy foods (e.g., carbohydrate-rich foods like rice and bread), and that leftover money was then spent on health foods (fruits and vegetables). Previous studies have also shown a relationship between self-reported food insecurity and low diet quality, particularly lower consumption levels of fruits and vegetables^(27,28)^. There have been efforts to improve food security and to increase the consumption of fruits and vegetables in Fiji, with support from the Government and Development Partners in providing plant seeds and gardening equipment for people to use in their home gardens^(29)^. Additionally, Leweniqila and Vunibola have published key recommendations for improving food security in Fiji by utilising Indigenous knowledge and traditional practices^(30)^. However, we did identify some conflicting ideas about the safety of eating home-grown foods, with men in one of the peri-urban focus groups concerned about the pollution of home-grown foods, including chemicals from the soil, while solutions for this were identified within the group, by using different/natural products on soil.

Generational changes were also evident, and these generational changes had relevance to gender equity. Firstly, both women and men across focus groups reflected that men are no longer planting and harvesting crops for food or fishing as much as their forefathers used to. Three reasons for this were provided: environmental change (climate change) making it harder to plant and grow foods, a lack of motivation to grow/catch food given foods can be purchased from a shop and because of the negative impacts of alcohol consumption. Kava is a traditional Fijian drink, often used during formal ceremonies and used for celebrations^(31)^. Kava, consumed on its own and in moderation, is thought to pose a minimal risk to human health^(32)^. However, women in the discussions reflected that men are now drinking alcohol along with kava and that they are drinking alcohol in excess. The negative health effects of consuming alcohol and kava collectively are highlighted by the Fijian Ministry of Health^(33)^. Our findings also highlight the negative social impacts, and potential impacts on food security, of this practice and emphasise the need for the consumption of alcohol and kava (collectively) to be addressed by health policy.

Participants’ relationships with their environments, and implications of climate change, were emphasised. Pacific Island countries are very minor contributors to greenhouse emissions; however, they are bearing the brunt of the climate crisis with rising sea levels and an increase in the frequency of storms and tropical cyclones^(34)^. Climate change was provided as another reason for the change in eating practices in comparison to participants’ forefathers, with a corresponding increased reliance on processed packaged foods. Previous research has depicted how women and men are, and will be, impacted differently by climate change^(35,36)^. Women, globally, are more at risk of being food insecure and of having malnutrition, and this is being exacerbated by climate change^(37)^. Climate change is a political priority for the Fijian Government^(38)^; however, climate change and the burden of poor diets are often viewed as separate issues, and therefore feature separately on political agenda^(39,40)^. There is a need to consider the overlapping relationship between climate change, food (particularly food security) and gender equity, both within Fiji and globally. These three factors and the cross-over between them are also central to the achievement of the sustainable development goals in Fiji^(41)^.

The school environment was identified as a key area of concern by participants. Both women and men in the rural focus groups reflected that confectionary was being sold in schools and kindergartens, by the teachers. Women in one of the peri-urban focus groups said that confectionary and sugary or salty snacks were being sold around their children’s’ schools by food hawkers. The parents and grandparents who raised these concerns reported feeling powerless; they felt that they did not have the power to tell the teachers to stop, and that they had to give their children or grandchildren money to buy confectionary otherwise they would refuse to go to school. Similar concerns have been raised in Samoa, where a key barrier to the implementation of their school food policy was the high prevalence of unhealthy food sold around schools^(42)^. Fiji has policies in this space, for example, the Fiji School Health Policy^(43)^ and the Policy on Food and School Canteens^(44)^; however, our findings highlight the need for more strongly implemented, and monitored, policies both in and around schools.

### Strengths and limitations

This is the first study to explore perceptions around healthy diets using an intersectional lens in Fiji. There are several limitations to our approach; firstly, we had a small convenience sample from four villages within the central district of Fiji. We visited villages during the working day, and in one of the villages not enough men were available to participate, so a different village was approached where men were able to participate. Given the size of the study, we were only able to focus on two gender identities within one ethnicity, and it is likely that people with other gender identities and other ethnicities have different perspectives of their food environment, which should be a focus of future research. There are also a number of important strengths to our study. We selected villages in rural and peri-urban locations to get an idea of different perspectives based on location. We took an intersectional approach for the interpretation of this work, which we believe is useful in this study to frame gender-related factors in a broader equity framework. We have identified several issues that may be considered the upstream causes or influences of gender-related inequities around nutrition and health in Fiji and propose these findings should be considered for policy formation going forward.

## Conclusion

Use of an intersectional framework aided the understanding of perceptions around healthy diets of iTaukei women and men in rural and semi-urban Suva, Fiji. Embedded traditional perceptions of gendered roles, responsibilities and beliefs around food and food provision were identified, along with important generational changes in food preferences and practices, and in experiencing the impacts of climate change. These findings highlight that food policy in Fiji needs to consider a range of different factors, both proximal and distal to individuals in order to improve diets equitably.

## Supplementary material

For supplementary material accompanying this paper visit https://doi.org/10.1017/S1368980022001677. click here to view supplementary material
