# Supplementary material for: Gender roles, generational changes and environmental challenges: an intersectional interpretation of perceptions on healthy diets among iTaukei women and men in Fiji
Source: Public Health Nutr. 2022 Aug 9;25(11):3146–57. doi: 10.1017/S1368980022001677 (PMC9991662; doi:10.1017/S1368980022001677)
Supplement: Supplementary file 1 [file S1368980022001677sup001.docx]

**Title: Gender roles, generational changes, and environmental challenges – an intersectional interpretation of perceptions on healthy diets among iTaukei women and men in Fiji**

**Supplementary table 1.** Discussion topics used in the focus groups (and translation to Fijian)

| Area of enquiry | Question/topic to discuss |
| --- | --- |
| Attitudes | How important is the food you eat to you?  *NA CAVA MADA NA YAGA NI KAKANA KO DAU KANIA ENA VEISIGA?* |
|  | What do you think about the quality of the food that you usually eat?  *NA CAVA NOMU NANUMA BALETA NA I VAKATAGEDEGEDE NI KAKANA KO KANIA ENA VEISIGA?* |
|  | Do you think your overall diet is different to what your grandparents ate when they were your age?  *NA CAVA NA KENA DUIDUI NI KAKANA KO DAU KANIA KEI NA KENA ERA DAU KANIA NA TUKADA SE O IRA NA NODA BUBU/NAU ENA GAUNA E LIU?* |
| Behaviours | What foods do you like eating?  *NA KAKANA CAVA SARA MADA KO DAU TALEITAKA MO KANIA?* |
|  | In your households, who normally sources/purchases the food?  *ENA NOMUDOU VUVALE, O CEI E DAU KAUTA MAI SE VOLIA NA KEMUDOU KAKANA?* |
|  | In your household, who normally prepares the meals?  *ENA NOMUDOU VUVALE, O CEI E DAU VAKARAUTAKA SE VAKASAQA NA KEMUDOU KAKANA?* |
|  | If you were to make a meal for your family, what would you make?  *KEVAKA MO VAKARAUTAKA SE VAKASAQARA NA KEMUDOU KAKANA, NA KAKANA CAVA KO NA VAKARAUTAKA?* |
|  | If you were to cook that meal but had the goal of making it “healthier” how would you do this?  *KEVAKA MO VAKASAQA SE VAKARAUTAKA NA KAKANA KO NANUMA TIKO MO VAKARAUTAKA, IA ME BULABULA CAKE MAI NA KENA VAKARAUTAKI ENA VEISIGA, O NA VEISAUTAKA BEKA VAKACAVA?* |
|  | What do you think about the food that members of your household eat?  *NA CAVA NOMU NANUMA ENA KAKANA KO DOU KANIA VAKAVUVALE ENA VEISIGA?* |
| Environment | What do you think about the food in your community?  *NA CAVA NOMU NANUMA ME BALETA NA KAKANA ENA NOMUDOU I TIKOTIKO SE KORO?* |
|  | What are your thoughts on the types of foods being sold around schools?  *NA CAVA NOMU NANUMA ENA MATAQALI KAKANA KA RA DAU VOLITAKI E KORONIVULI SE NA KENA ERA DAU VOLITAKI VOLEKATA NA KORONIVULI?* |
|  | Can you get the foods that you wish to get when you want them?  *E RAWA BEKA NI KO NA KANIA SE KAUTA MAI NA KAKANA KO DAU GADREVA SE TATADRATAKA ENA GAUNA KO VINAKATA KINA?* |
|  | How available is fresh food in your community?  *E TU VAKA RAWARAWA BEKA NA KAKANA KA SEGA NI VOLI MAI NA SITOA (FRESH) ME VAKA NA VUATA, DRAUNIKAU, LEWE NI MANUMANU SE SASALU NI WAITUI?* |
| Knowledge | What do you see as the main health risks to yourself and your family?  *NA CAVA KO NANUMA NI RAWA NI TOKONA SE VAKAVU TAUVIMATE VEI IKO KEI RATOU NA LEWE NI NOMU VUVALE?* |
|  | As a (woman or man, depending on the group) do you have any additional or different health concerns?  *E TIKO TALE BEKA E DUA NA KA KO NANUMA NI RAWA NI TOKONA NA TAUVIMATE ENA NOMUDOU VUVALE ENA NOMU RAI VAKA MARAMA (SE TURAGA)?* |
|  | How would you describe a “healthy” body shape for a man?  V*AKAMACALATAKA MADA NAI RAIRAI SE TUVAKI NI DUA NA TURAGA BULABULA VINAKA?* |
|  | How would you describe a “healthy” body shape for a woman?  *VAKAMACALATAKA MADA NAI RAIRAI SE TUVAKI NI DUA NA MARAMA BULABULA VINAKA?* |
|  | What role do you think diet (what you eat) has in your health?  *NA CAVA NA I TAVI NI KAKANA (KO DOU KANIA ENA VEISIGA) KINA NOMU BULA?* |
|  | What specific health issues can be addressed through the food that you eat?  *NA MATAQALI MATE CAVA SARA MADA KO NANUMA NI RAWA NI WALI ENA KAKANA EDA KANIA?* |
|  | Where do you find information on the food that you eat?  *E VEI KO RAWA NI VULICA SE KILA KINA NA I TUKUTUKU NI KAKANA KO KANIA?* |
|  | As a (women or man, depending on the group), do you have any additional or different diet/nutrition requirements?  *E DUIDUI BEKA NA KAKANA SE NA NUTRIENTS E GADREVA E DUA NA TURAGA (SE MARAMA)?* |
